# Hepatitis C Virus Reactivation in Anti-HCV Antibody-Positive Patients with Chronic Hepatitis B Following Anti-HBV Therapies

**DOI:** 10.3390/v14091858

**Published:** 2022-08-24

**Authors:** Yi-Tse Su, Ming-Ling Chang, Rong-Nan Chien, Yun-Fan Liaw

**Affiliations:** 1 Division of Hepatology, Department of Hepatology and Gastroenterology, Chang Gung Memorial Hospital, Taoyuan 333423, Taiwan; 2 Department of Medicine, College of Medicine, Chang Gung University, Taoyuan 333323, Taiwan

**Keywords:** HBV, CHC, past HCV infection, co-infection, nucleos(t)ide analogue

## Abstract

Background and Aims: Whether hepatitis C virus (HCV) reactivation occurs and how the viral load evolves in anti-HCV antibody-positive chronic hepatitis B (CHB) patients who underwent nucleos(t)ide analogue (Nuc) therapies remain unsolved. Methods: A cohort of 66 such patients was studied. Results: At the start of Nuc treatment (baseline), all patients had detectable hepatitis B virus (HBV) DNA levels (6.05 ± 1.88 log IU/mL), while HCV RNA levels (3.79 ± 1.43 log IU/mL) were detected (i.e., chronic hepatitis C (CHC)) in only 13 patients (19.7%). Following Nuc therapies, HBV DNA levels reached the nadirs at end of therapy (EOT) (6.05 ± 1.88 vs. 0.25 ± 0.99 log IU/mL, *p* < 0.0001) and relapsed at 6 months after EOT (6mEOT) at a level of 3.45 ± 2.64 log IU/mL compared with EOT (*p* < 0.0001). Among the 13 CHC patients, a non-significant decrease in HCV RNA was noted at EOT (3.52 ± 1.71 vs. 2.77 ± 2.63 log IU/mL, *p* = 0.166) but tended to decrease further at 6mEOT (2.77 ± 2.63 vs. 1.89 ± 2.06 log IU/mL, *p* = 0.063). Two of the thirteen CHC patients showed an increase in HCV-RNA ≥ 1 log10 IU/mL at EOT, and one of the fifty-three patients with undetectable HCV RNA at baseline (i.e., resolved past HCV infection) showed detectable HCV RNA at year 1 (3200 IU/mL) and year 2 (1240 IU/mL) following entecavir therapy. Conclusions: HCV reactivation did occur during HBV suppression, and the rate was 4.5% (3/66), 15.4% (2/13), and 1.9% (1/53), for all patients, CHC patients, and patients with resolved past HCV infection, respectively. The reverse HBV and HCV viral evolutions at 6mEOT indicate that HBV relapse may suppress HCV replication again.

## 1. Introduction

Concurrent hepatitis B virus (HBV) and hepatitis C virus (HCV) infection is not uncommon in highly endemic areas of HBV infection [1]. It is a complex clinical entity that has an estimated worldwide prevalence of up to 15% [2]. The reported series on the seroprevalence of HCV indicates that HCV is found in >10% of patients infected with HBV worldwide [3]. Higher risks of liver-related complications, such as liver cirrhosis and hepatocellular carcinoma (HCC), are found in patients dually infected with HBV and HCV than those with either infection alone [4]. The primary goal of the treatment for concurrent HCV and HBV infection is to eliminate or permanently suppress both viruses, with the dominant virus being identified and treated first [5]. On the one hand, HCV is usually the dominant virus [4] as HBV gene expression and replication are suppressed by its core protein [6]; on the other hand, although it has been less frequently observed, reciprocal suppression is also possible [4], especially in cases of acute HBV superinfection in chronic HCV infection [7]. HBV reactivation has been reported in patients with concurrent HBV and HCV infection following either direct-acting antiviral (DAA) [8] or interferon-based anti-HCV therapy [1,9]. In contrast, HCV reactivation is rarely reported in patients concurrently infected with HBV and HCV following anti-HBV therapy. However, an episode of HCV reactivation in a dually infected patient who achieved an HBV viral response following lymphoblastoid interferon therapy has been noted [10]. How HBV and HCV evolve and whether HCV reactivates in dually infected patients who receive nucleos(t)ide analogue (Nuc) anti-HBV therapies remain unsolved. The natural course of HCV viral evolution in patients concurrently infected with HBV and HCV can hardly be observed nowadays because of almost universal HCV clearance by potent direct-acting antiviral (DAA) administration in patients with chronic hepatitis C (CHC) [11]. Therefore, we constructed a retrospective cohort of Nuc-treated chronic hepatitis B (CHB) patients who were seropositive for anti-HCV antibody (Ab) to examine the viral evolutions in such dually infected patients.

## 2. Materials and Methods

### 2.1. Patients

Adult patients with chronic hepatitis B (CHB) (>18 years old) seropositive for anti-HCV Ab who had been treated with Nucs were consecutively enrolled from October 1999 to October 2021 at a tertiary care center. CHB was defined as positive for hepatitis B surface antigen (HBsAg) > 6 months. CHC was defined as detectable HCV RNA by PCR for >24 weeks, and resolved past HCV infection was defined as seropositive for anti-HCV Ab but with undetectable HCV RNA [11]. HCV reactivation was defined as an increase in HCV-RNA ≥ 1 log10 IU/mL over baseline [12]. Subjects with other causes of liver diseases (including human immunodeficiency virus (HIV), delta virus, and bacterial infections), alcoholism, autoimmune liver diseases, or malignancy were excluded. To avoid interfering with HCV RNA evolution, patients with previous anti-HCV therapy, including interferon-based or DAA-based therapy, were also excluded. The diagnosis of liver cirrhosis was made by earlier or recent histologic findings or ultrasonographic findings compatible with cirrhosis and supplemented with clinical findings such as thrombocytopenia, splenomegaly, or esophageal or gastric varices [13]. A hepatitis flare was defined as an abrupt increase in serum alanine aminotransferase (ALT) > 5× the normal upper limit (36 U/L) [14]. Hepatic decompensation was defined as the presence of jaundice, coagulopathy, and/or development of ascites/encephalopathy [15]. HCC was diagnosed by histology/cytology or sonographic findings plus a high alpha-fetoprotein (AFP) level or imaging findings as described in generally accepted guidelines [13]. The decompensated patients were hospitalized and treated with Nucs in addition to conventional supportive measures, and they were closely monitored. The patients who initially started with lamivudine (LAM) and then developed resistance were rescued by the early administration of adefovir or were switched to entecavir (ETV) or tenofovir disoproxil fumarate (TDF). The patients who initially started with ETV and then developed resistance were switched to TDF or tenofovir alafenamide (TAF). The CHB patients ceased Nuc therapy according to the Asian Pacific Association for the study of the Liver (APASL) stopping rules [16] at the discretion of a joint physician/patient decision considering disease severity and reimbursement issues.

### 2.2. Methods

Baseline assessments included: age, sex, ALT, AFP, total bilirubin (bili-t), international normalized ratio (INR) of prothrombin time, albumin, creatinine, platelet, HBV DNA, hepatitis B e antigen (HBeAg), HBV genotype, anti-HCV and anti-HDV Abs. HCV RNA, and HIV Ag/Ab combination tests were performed. Liver biochemical tests were performed at the clinical pathology laboratories of the hospital using routine automated techniques. The HBV genotype was determined using PCR-restriction fragment length polymorphism of the surface gene of HBV. The serum HBV DNA level was measured using the hybrid Capture II assay (Digene Corp, Gaithersburg, MD; lower limit of detection, 1.4 × 10^5^ copies/mL) before April 2007. Thereafter, automated quantitative polymerase chain reaction assays (Roche Diagnostics, Pleasanton CA; Cobas Amplicor HBV Monitor; lower limit of detection, 300 copies/mL or the Roche COBAS TaqMan HBV Test; lower limit of detection, 12 IU/mL) were used for HBV DNA quantifications. Stored serum samples were assayed retrospectively when data were missing. Since the Digene assay reports the HBV DNA level in copies/mL, we divided the levels by a factor of 5.6 to report the IU/mL in this study. The HCV RNA levels were tested using the COBAS Amplicor (Roche Diagnostics, Tokyo, Japan; lower limit of detection: 15 IU/mL, specificity: 100%). Anti-HCV Ab was assayed using a commercial third-generation enzyme immunoassay (Axsym HCV, version 3; Abbott Diagnostics, North Chicago, IL, USA). Anti-HDV Ab was assayed using radioimmunoassay kits (Abbott Diagnostics, North Chicago, IL, USA). ALT, AFP, and bili-t levels and sonography were assessed every 3–6 months; HBV DNA and HCV RNA levels were assayed as soon as HBV- or HCV-associated hepatitis or flare was suspected. At the end of Nuc therapy (EOT), and 6 months after EOT (6mEOT), both HBV DNA and HCV RNA were measured for all the enrolled patients.

### 2.3. Statistical Analysis

All statistical analyses were performed using the Statistical Package for the Social Sciences (SPSS) software (version 21.0, SPSS Inc., Chicago, IL, USA) or GraphPad Software (GraphPad Prism version 8.0.0 for Windows, GraphPad Software, San Diego, CA, USA). To compare the different variables between groups, continuous variables were analyzed using Student’s t-test or the Mann–Whitney U test, while categorical variables were analyzed using a chi-squared test or Fisher’s exact test when appropriate. Continuous variables are summarized as the means ± standard deviation (SD), and categorical variables are indicated as frequencies and percentages. A paired t-test or a Wilcoxon matched-pairs signed rank test was used with the same variables in the same individuals. Kaplan–Meier analyses were used to assess the cumulative incidences of various events. Statistical significance was defined at the 5% level based on two-tailed tests of the null hypothesis.

### 2.4. Institutional Review Board Approval

The study was conducted in accordance with good clinical practice and all applicable regulations, including the Declaration of Helsinki and local regulatory requirements, and it was approved by the ethics committee.

## 3. Results

### 3.1. Baseline Characteristics

The baseline characteristics of the enrolled patients are shown in Table 1. Of 66 CHB patients, 40 (60.6%) were males, the mean age was 52.1 years, 15 (22.7%) were HBeAg-positive, 51 (77.3%) were infected with genotype B and 15 (22.7%) with genotype C HBV, and the mean ALT level was 362.3 U/L. All 66 patients had detectable HBV DNA levels (6.05 ± 1.88 log IU/mL), and 13 of them (19.7%) had detectable HCV RNA levels at a mean level of 3.79 log IU/mL and were classified as having CHC, whereas the remaining 53 (80.3%) patients were classified as resolved past HCV infection. Compared with patients with resolved past HCV infection, CHC patients had higher rates of LAM therapy and genotype B HBV infection (100% vs. 71.7%, *p* = 0.0298) and lower genotype C HBV infection (0 vs. 28.3%, *p* = 0.0298). No significant differences in any other investigated baseline profiles including ALT levels were noted between these two groups. In addition, patients infected with genotype B or C HBV had similar HBV DNA levels (6.028 +/−1.837 vs. 5.959 +/−2.052 log IU/mL, *p* = 0.9).

**Figure 1:**
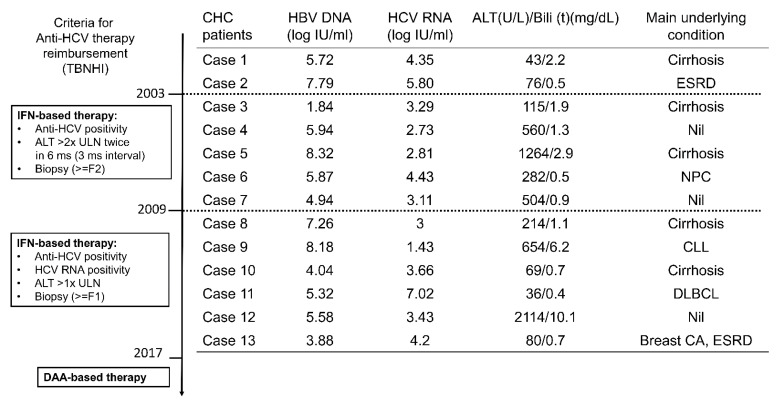
The anti-HCV therapy reimbursement policy of the Taiwan Bureau of National Health Insurance (TBNHI) and the baseline characteristics of the 13 CHB patients with CHC. IFN: interferon; ULN: upper limit of normal; ms: months; F1: fibrosis score 1; F2: fibrosis score 2; ESRD: end-stage renal disease (both patients with ESRD had undergone hemodialysis); NPC: nasopharyngeal carcinoma; CLL: chronic lymphocytic leukemia; DLBCL: diffuse large B-cell lymphoma; Nil: nothing; CA: cancer. Case 13 received the DAA therapy in 2019 and acquired an SVR after the therapy.

In patients concurrently infected with HBV and HCV, it is generally agreed that the treatment should be aimed toward the dominant virus [5]. The anti-HCV therapy reimbursement policy of the Bureau of National Health Insurance (BNHI) of the country was set up in 2003 and revised in 2009 and in 2017. The 13 CHB patients with CHC were enrolled from 2001 to 2016 (Figure 1) when interferon-based therapy (for patients with liver biopsy-documented hepatic fibrosis) but not DAA therapy was reimbursed by Taiwan BNHI. All 13 patients, except 2, were HBV dominant according to their baseline viral loads. Of the two patients with HCV dominance, one had lymphoma and the other had breast cancer. Anti-HCV therapy was not prescribed due to concerns of drug–drug interaction between interferon-based therapy and anti-cancer therapy and interferon-associated immune modulation. The anti-cancer medications were as follows: cisplatin, etoposide, mitomycin, and epirubicin for patient 6 (with nasopharyngeal carcinoma); no anti-cancer medication for patient 9 (with chronic lymphocytic leukemia) during the enrollment period; rituximab, cyclophosphamide, doxorubicin, and vincristine for patient 11 (with diffuse large B-cell lymphoma); and no anti-cancer medication for patient 13 (with breast cancer) during the enrollment period. Of the 11 patients with HBV dominance, the interferon-based anti-HCV therapy was either not reimbursed (two patients enrolled before 2009, and five patients enrolled after 2009 but refused to receive liver biopsy) or not suitable due to hepatic decompensation (four patients). Thus, all 13 CHC patients did not receive any anti-HCV therapy during the observation period.

### 3.2. The Evolutionary Trends of HBV and HCV Viral Loads

**Figure 2:**
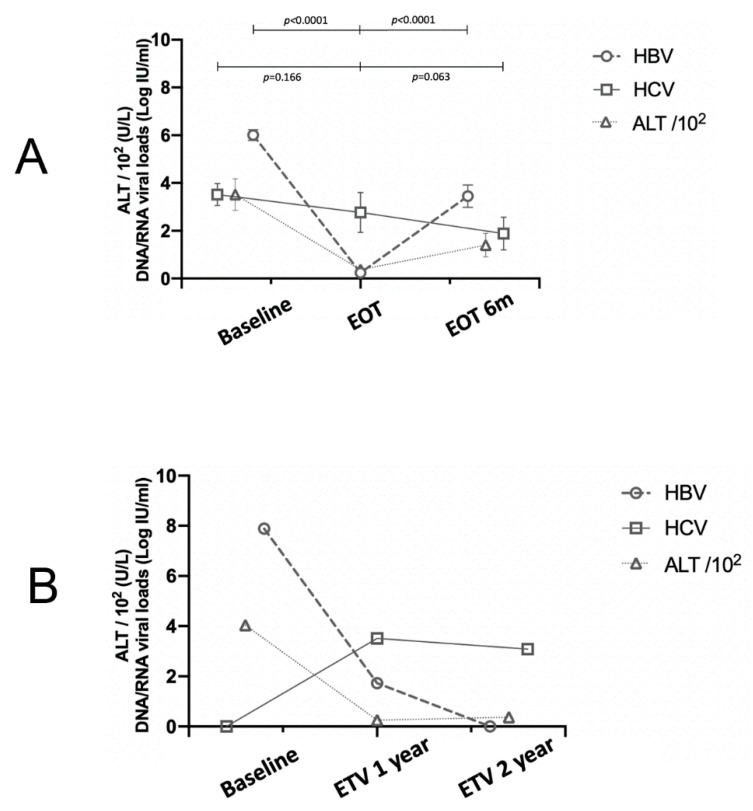
(**A**) HBV DNA, HCV RNA, and ALT level evolutions among the 68 CHB patients. EOT: end of therapy, EOT 6 m: 6 months after EOT. (**B**) HBV DNA, HCV RNA and ALT level evolutions of HCV reaction in a patient with previously resolved HCV infection. ETV: entecavir.

The 66 CHB patients had been treated with Nucs for a mean duration of 46.2 months and followed up for a mean duration of 84.8 months. For all patients, the HBV DNA levels decreased from 6.05 ± 1.88 log IU/mL at pretherapy baseline to 0.25 ± 0.99 log IU/mL at EOT (*p* < 0.0001) and increased after cessation of therapy to 3.45 ± 2.64 log IU/mL at 6mEOT (*p* < 0.0001). Among the 13 CHC patients, no significant HCV RNA decline was noted from 3.79 ± 1.43 at baseline to 2.77 ± 2.63 log IU/mL at EOT (*p* = 0.166) but tended to decrease further at 6mEOT (when HBV relapsed to 3.45 ± 2.64 log IU/mL), from 2.77 ± 2.63 to 1.89 ± 2.06 log IU/mL (*p* = 0.063). The longitudinal evolution of HBV DNA and HCV RNA levels crossed over after EOT (Figure 2A). Specifically, 2 of the 13 CHC patients showed an increase in HCV-RNA ≥ 1 log10 IU/mL at EOT compared with baseline (Table 2) (a 36-year-old male with dyslipidemia treated with ETV and a 63-year-old male with cirrhosis treated with LAM), and 1 with resolved past HCV infection (baseline HBV DNA: 7.89 logs IU/mL) showed detectable HCV RNA levels at year 1 (HBV DNA: 54 IU/mL; HCV RNA: 3200 IU/mL) and year 2 (HBV DNA: undetected; HCV RNA: 1240 IU/mL) of ETV therapy (Figure 2B) (a 68-year-old male patient with cirrhosis and hypertension). Normal or mild ALT elevation was noted upon these HCV reactivations, ranging from 27 to 62 U/L. Thus, the HCV reactivation rate among the 66 patients, the 13 CHC patients, and the 53 patients with resolved past HCV infection was 4.5% (3/66), 15.4% (2/13), and 1.9% (1/53), respectively. 

### 3.3. Long-Term Outcomes

As shown in Table 3, 6 (9.1%) of the 66 CHB patients developed HCC from 3 to 79 months after baseline and 7 (10.6%) died (3 of HCC, 3 of decompensation, and 1 of cardiovascular event) from 23 to 79 months after baseline. Of the three cases with HCV reactivation, one with resolved past HCV infection died of HCC. This patient suffered from HCC after treatment with ETV for 6 months; the HCC progressed despite treatments of radiofrequency ablation and transcatheter arterial chemoembolization, and the patient expired after treatment with ETV for 43 months. None of the three patients with HCV reactivation experienced hepatitis flare, hepatic decompensation, or mortality during HCV reactivation. Compared with patients with resolved past HCV infection, the CHC patients had a higher rate (30.8 vs. 5.7%, *p* = 0.0239) and cumulative incidence of mortality (35.7 vs. 7.5%, *p* = 0.0173).

## 4. Discussion

The most compelling findings of the current study are as follows: (1) HCV reactivation did occur during HBV suppression, and the rate was 15.4% in CHB patients with CHC and 1.9% in CHB patients with resolved past HCV infection; (2) the reverse viral load evolutions of HBV and HCV at 6mEOT indicate that HBV relapse may suppress HCV replication again; (3) among CHB patients seropositive for anti-HCV Ab who require anti-HBV therapy, approximately 20% had detectable HCV viremia, of which none were infected with genotype C HBV; (4) patients with CHC had a higher mortality rate than that of patients with a resolved past HCV infection.

HBV reactivation during/after successful HCV therapy has been well-documented in patients with dual HBV/HCV infections [1,8,9]. Notably, to the best of our knowledge, the current study is the first report of HCV reactivation during successful HBV suppression with Nuc therapy. The pooled proportion of HBV reactivation was 24% of CHC patients with CHB but only 1.4% of CHC patients with resolved HBV infection following DAA therapy [8]; likewise, the rate of HCV reactivation was 15.4% of CHB patients with CHC but only 1.9% of CHB patients with resolved past HCV infection following Nuc therapy. Thus, a resolved viral infection, regardless of viral type, is associated with a lower reactivation rate in HBV/HCV dual infection. Moreover, the lower HCV reactivation rate (15.4%) following HBV suppression compared to the HBV reactivation rate (24%) following HCV suppression among the patients with chronic dual infection echoes the fact that HBV suppresses HCV less effectively [4]. These phenomena suggest that HBV and HCV have a reciprocal suppressive effect, though HCV is usually the dominant one [17]. Although all three cases with HCV reactivation were not prescribed with any anti-HCV therapy, none had ever experienced hepatitis flare, decompensation, or mortality during HCV reactivation. By contrast, fulminant hepatic failure might occur due to HBV reaction in dually infected patients following DAA therapy [18]. This is probably because HCV hijacks cellular mechanisms to suppress the innate immune response and hepatitis activity and then gains the survival advantage [19]. However, caution might be necessary for potentially severe complications after HCV reactivation in patients with comorbidities as hepatitis C with hepatic failure has been reported in elderly patients [20], patients with cirrhosis [21], young patients who underwent steroid pulse therapy [22], and patients after the withdrawal of chemotherapy [23]. In these vulnerable patients with resolved past HCV infection following anti-HBV therapy, HCV RNA levels might be monitored upon deteriorated liver function. Moreover, although all the enrolled patients had received anti-HBV therapy, further studies are required to confirm whether HCV reactivation only occurs in CHB patients with anti-HBV therapy. Particularly, HCV exerts a suppressive effect on HBV and may enhance the seroclearance of HBV antigens or even usurp the role of the preexisting virus as the agent for continuing hepatitis [24]. The suppressive effect is achieved by the transcription of HBV RNAs being repressed by HCV core proteins [6,25]. However, some in vitro studies show that the direct interferences between HBV and HCV are negligible [26,27], and altered immune responses [28] induced by HCV infection, including hepatic interferon (IFN) response [29], might account for the suppressive effect of HCV on HBV replication. Also, HBV might evade the induction of IFN and IFN-induced antiviral effects [30], and HBV infection does not rescue HCV from the IFN-mediated response [31]. Moreover, miR-122 stimulates HCV replication but suppresses HBV replication [32]. All the intricate viral interactions explain why a wide spectrum of HCV and HBV virological patterns may occur in cases of coinfection [33]. For example, during and after DAA-based treatment, among HBsAg-positive patients, the HBV reactivation rate varied and ranged from 2% to 57% [9]. In the CHC cases with acute HBV superinfection, HCV infection might be completely resolved and CHB developed [7], or both HBV and HCV infections may be recovered. This suggests that HBV, as the newcomer, may also suppress the pre-existing HCV [7,34]. In HBV-endemic countries with perinatal transmission as the major route of infection such as Taiwan [35], dual infection of HBV and HCV mostly occur as HCV superinfection over pre-existing HBsAg carriage [36]. Thus, HCV rather than HBV was the newcomer that accounted for most cases in the current study. Similar to patients mono-infected with HBV treated with Nucs, most patients exhibited HBV DNA elevation at 6mEOT. Interestingly, a decreasing trend of HCV RNA levels was noted at 6mEOT when HBV relapsed. The reverse HBV and HCV viral load evolutions at 6mEOT suggest that HBV relapse might suppress HCV replication again.

Among anti-HCV-Ab-positive patients who never received any anti-HCV therapy, HCV RNA-positive rates range from 54% [37] to 92.5% [38], which are much higher than that (19.7%) noted in the current study involving CHB patients with high HBV DNA levels requiring anti-HBV therapy. Again, this supports the possibility that highly replicative HBV might suppress or even expel HCV. Of note, a higher genotype B HBV infection rate was noted in CHC patients than in those with resolved past HCV infection. Indeed, none of the former were infected with genotype C HBV. Compared with genotype B cases, patients with genotypes C HBV infection have higher HBV viral loads but lower rates of spontaneous HBeAg seroconversion [39]. Although the genotype B and C cases had similar HBV DNA levels, the HBeAg-positive rates and HBV DNA levels of the CHC patients and patients with resolved past HCV infection were not different, the fact that none of the CHC patients were genotype C cases potentially indicates a higher suppressive effect on HCV replication of genotype C than genotype B HBV. In addition, the CHC patients had a higher mortality rate than those with resolved past HCV infection, which is consistent with the observation that among the patients with the dual infection involving HCV, those with co-replication of viruses tend to have more severe and progressive liver diseases compared with patients with a single-virus infection [1,3,4].

There are some limitations to the current study. First, because of the retrospective nature of the study database, a thorough assessment regarding the HBV mutations, HCV genotype, and the immunological basis of the viral interaction could not be performed on the patients enrolled in the early years, and the antiviral regimens could not be uniformly administered. Second, the limited case number might lead to a statistical type II error. Third, two patients had been prescribed anti-cancer medications, which might have affected the patients’ immunity and viral evolution. Fourth, given that the lower detection limit of HCV RNA was 15 IU/mL, HCV reactivation in patients with a viral load lower than 15 IU/mL cannot be detected, while extracting large volumes of plasma/whole blood or concentrating viral particles with ultracentrifugation of serum before PCR might increase the detection sensitivity of HCV RNA [40]. Fifth, the data from a study conducted in a single center in Taiwan might not be generalized to other CHB patients of different ethnicities or countries, or patients infected with HBV genotypes other than genotype B or C. Further large randomized controlled studies with uniform medications, comprehensive immunological surveys, and sensitive HCV RNA assays such as ultracentrifugation of serum before PCR in indicated cases are required to verify the associated findings described herein.

Taken together, among CHB patients seropositive for anti-HCV Ab, HCV reactivation did occur during HBV suppression in 15.4% of the CHC patients and 1.9% of the patients with resolved past HCV infection. HBV relapse after cessation of Nuc may again suppress the reactivated HCV. Together with HBV reactivation during antiviral therapy for HCV infection, the results of the current study confirm the reciprocal HBV/HCV suppressive effect.

## Figures and Tables

**Table 1:** Baseline characteristics of the enrolled CHB patients seropositive for anti-HCV Ab.

	All (*n* = 66)	CHC (*n* = 13)	Resolved Past HCV Infection (*n* = 53)	*p* Values
Age (years) *	52.1 ± 10.9 (51.5; 32–76)	52.5 ± 14.0 (53; 35–71)	51.9 ± 10.2 (51; 32–76)	0.8796
Male, *n* (%)	40 (60.6)	7 (53.8)	33 (62.3)	0.7526
HBV genotype, *n* (%)				
B	51 (77.3)	13 (100)	38 (71.7)	0.0298
C	15 (22.7)	0	15 (28.3)	0.0298
HBeAg positivity, *n* (%)	15 (22.7)	3 (23.1)	12 (22.6)	>0.9999
Cirrhosis, *n* (%)	33 (50.0)	5 (38.5)	28 (52.8)	0.5372
Decompensation, *n* (%)	2 (3.0)	0 (0)	2 (3.8)	0.4769
HBV DNA (log IU/mL) *	6.05 ± 1.88 (5.94; 1.84–9.82)	5.75 ± 1.86 (5.72; 1.84–8.32)	6.13 ± 1.89 (6.2; 2.04–9.82)	0.5155
HCV RNA (log IU/mL) *	0.75 ± 1.64 (0; 0–7.02)	3.79 ± 1.43 (3.42; 1.43–7.02)	undetectable	<0.0001
ALT (U/L) *	362.3 ± 545.5 (142; 19–2735)	494.7 ± 624 (248; 36–2114)	332.4 ± 528.2 (129; 19–2735)	0.3561
Bili (t) (mg/dL) *	2.2 ± 3.57 (0.95; 0.4–22)	2.39 ± 2.92 (1.2; 0.4–10.1)	2.15 ± 3.73 (0.9; 0.4–22)	0.8371
INR *	1.20 ± 0.23 (1.2, 1–2.1)	1.17 ± 0.21 (1.1; 1–1.4)	1.2 ± 0.23 (1.2; 1–2.1)	0.7951
Albumin (g/dL) *	3.92 ± 0.68 (4.05; 2.3–4.9)	3.97 ± 0.59 (4.2; 2.7–4.8)	3.91 ± 0.71 (4; 2.3–4.9)	0.8033
AFP (ng/mL) *	66.6 ± 380.1 (5; 2–3031)	56.9 ± 157.7 (5; 3–556)	68.7 ± 415.4 (5.3; 2–3031)	0.9232
Platelet (10^3^/uL) *	175 ± 64.9 (174.5; 38–342)	178.4 ± 64.9 (189; 85–279)	174.3 ± 65.5 (174; 38–342)	0.8558
Creatinine (mg/dl) *	1.02 ± 0.87 (0.86; 0.5–7)	1.05 ± 0.40 (0.99; 0.54–2)	1.01 ± 0.95 (0.8; 0.5–7)	0.8975
Nucs, *n* (%)				
Ldt	1 (1.5)	0 (0)	1 (1.9)	>0.9999
LAM	15 (22.7)	7 (53.8)	8 (15.1)	0.0065
ETV	40 (60.6)	5 (38.5)	35 (66.0)	0.1115
TDF	8 (12.1)	1 (7.7)	7 (13.2)	>0.9999
TAF	2 (3.0)	0 (0)	2 (3.8)	>0.9999

CHB: chronic hepatitis B; HCV: hepatitis C virus; CHC: chronic hepatitis C; HBV: hepatitis B virus; HBeAg; hepatitis B e antigen; ALT: alanine aminotransferase; bili (t): total bilirubin; INR: international normalized ratio; AFP: alpha-fetoprotein; Nuc: nucleos(t)ide analogue; Ldt: telbivudine; LAM: lamivudine; ETV: entecavir; TDF: tenofovir disoproxil fumarate; TAF: tenofovir alafenamide; *: mean +/− standard deviation (median; range).

**Table 2:** Viral load evolution of HCV reactions of two CHC patients.

		Baseline	EOT	6mEOT	Underlying Conditions
Case 1	HCV RNA (logIU/mL)	3.42	5.85	5.31	Dyslipidemia
	HBV DNA (logIU/mL)	5.58	undetectable	undetectable	
	ALT (U/L)	2114	49	62	
Case 2	HCV RNA (logIU/mL)	2.81	5.20	3.59	Cirrhosis
	HBV DNA (logIU/mL)	8.32	undetectable	2.68	
	ALT (U/L)	1264	27	28	

HCV: hepatitis C virus; CHC: chronic hepatitis C; HBV: hepatitis B virus; ALT: alanine aminotransferase; EOT: end of treatment; 6mEOT: 6 months after the end of treatment.

**Table 3:** Follow-up/treatment durations and outcomes of the enrolled patients.

	All (*n* = 66)	CHC (*n* = 13)	Resolved Past HCV Infection (*n* = 53)	*p* Values
Follow-up duration (m) *	84.8 ± 53.6 (79.5; 8–213)	99.0 ± 66.4 (79; 25–213)	81.3 ± 50.1(80; 8–193)	0.2903
Treatment duration (m) *	46.2 ± 42.1 (36; 5–177)	37.5 ± 51.9 (14; 5–177)	48.3 ± 39.7(36; 8–161)	0.4117
Outcomes				
Mortality, *n* (%)	7 (10.6)	4 (30.8)	3 (5.7)	0.0239
HCC, *n* (%)	6 (9.1)	2 (15.4)	4 (7.5)	0.3367

CHB: chronic hepatitis B; CHC: chronic hepatitis C; m: months; HCC: hepatocellular carcinoma; *: mean +/− standard deviation (median; range).

## Data Availability

The datasets used and/or analyzed during the current study are available from the corresponding author on reasonable request. Patient consent was waived due to retrospective nature of this study, along with no modifications in patient management, and all personal information was encrypted in a database, patient data were anonymized. There was no breach of privacy.

## Abbreviations

HCV: hepatitis C virus; CHB: chronic hepatitis B; Nuc: nucleos(t)ide analog; HBV: hepatitis B virus; CHC: chronic hepatitis C; EOT: end of therapy; 6mEOT: 6 months after EOT; HCC: hepatocellular carcinoma; Ab: antibody; HBsAg: hepatitis B surface antigen; DAA: direct antiviral agent; HBeAg; hepatitis B e antigen; ALT: alanine aminotransferase; bili (t): total bilirubin; INR: international normalized ratio; AFP: alpha-fetoprotein; Nuc: nucleos(t)ide analog; Ldt: telbivudine; LAM: lamivudine; ETV: entecavir; TDF: tenofovir disoproxil fumarate; TAF: tenofovir alafenamide; APASL: Asian Pacific Association for the study of the Liver; SPSS: Statistical Package for the Social Sciences; SD: standard deviation; IFN: interferon.
